# Feeding Deterrents against Two Grain Storage Insects from *Euphorbia fischeriana*

**DOI:** 10.3390/molecules16010466

**Published:** 2011-01-10

**Authors:** Zhu Feng Geng, Zhi Long Liu, Cheng Fang Wang, Qi Zhi Liu, Sheng Min Shen, Zi Mu Liu, Shu Shan Du, Zhi Wei Deng

**Affiliations:** 1 Analytical and Testing Center, Beijing Normal University, Beijing 100875, China; 2 Department of Entomology, China Agricultural University, Haidian District, Beijing 100094, China; 3 Protection and Utilization of Traditional Chinese Medicine of Beijing Area Major Laboratory, Beijing Normal University, Haidian District, Beijing 100875, China

**Keywords:** feeding deterrents, *Euphorbia fischeriana*, *Tribolium castaneum*, *Sitophilus zeamais*

## Abstract

The screening of several Chinese medicinal herbs for insecticidal principles showed that *Euphorbia fischeriana* roots possessed significant feeding deterrent activity against two stored-product insects (*Tribolium castaneum* and *Sitophilus zeamais*). From ethanol extract, four feeding deterrents were isolated by bioassay-guided fractionation. The compounds were identified as jolkinolide B, 12-deoxyphorbol 13-(9*Z*)-octadecenoate 20-acetate, 17-hydroxyjolkinolide A and B on the basis of their phytochemical and spectral data. Jolkinolide B and 17-hydroxyjolkinolide B possessed strong feeding deterrent activities against *S. zeamais* (EC_50 _= 342.1 and 543.9 ppm, respectively) and *T. castaneum* adults (EC_50_ = 361.4 and 551.5 ppm, respectively). 17-Hydroxyjolkinolide A and 12-deoxyphorbol 13-(9*Z*)-octadecenoate 20-acetate A also exhibited feeding deterrent activity against the two grain storage insects with EC_50_ values of 631.9 and 884.3 ppm for *S. zeamais* and 656.5 and 1058.4 ppm for *T. castaneum* adults.

## 1. Introduction

Botanical pesticides have the advantage of providing novel modes of action against insects that can reduce the risk of cross-resistance as well as offering new leads for design of target-specific molecules [1,2]. During a screening program for new agrochemicals from Chinese medicinal herbs and wild plants, *Euphorbia fischeriana* Steud. roots (Family: Euphorbiaceae) were found to possess significant feeding deterrence activity against two stored-product insects, red flour beetles (*Tribolium castaneum* Herbst) and maize weevil (*Sitophilus zeamais* Motsch.). *E. fischeriana* is a perennial herbaceous plant with milky juice, distributed mainly in north China [3]. *S. zeamais* and *T. castaneum* are the most widespread and destructive primary insect pests of stored cereals [4]. Infestations not only cause significant losses due to the consumption of grains; they also result in elevated temperature and moisture conditions that lead to an accelerated growth of molds, including toxigenic species [5]. Control of stored product insects relies heavily on the use of synthetic insecticides and fumigants, which has led to problems such as disturbances of the environment, increasing costs of application, pest resurgence, pest resistance to pesticides and lethal effects on non-target organisms in addition to direct toxicity to users [6]. These problems have highlighted the need for the development of new types of selective stored product pest-control alternatives.

The dried plant roots of *Euphorbia fischeriana*, known as “lang-du” in traditional Chinese medicine, have long been used for the treatment of a wide range of ailments, including edema, ascites, ingestion, as well as liver and lung cancer [7,8,9]. The ethanol extracts of *E. fischeriana* roots were found to possess toxicity against *Oncomelania* snails and miracidiums of *Schistosoma* [10]. Aqueous extracts of *E. fischeriana* roots have been used to control aphids (*Aphis gossypii*), cabbage beetle (*Colaphellus bowringi*) and *Pieris rapae* on vegetables and rice leaf roller *Cnaphalocrocis medinalis* as well as spider mites [11]. Petroleum ether extracts of *E. fischeriana* roots possessed strong contact toxicity against carmine spider mite, *Tetranychus cinnabarinus* adults [12] and eggs [13]. Moreover, one formulation based on ethanol extracts of *E. fischeriana* roots had been evaluated in control of cabbage moth, *Barathra brassicae* and green peach aphids, *Myzus persicae* [14]. 

Due to the fact that it is a common Chinese herb used in medicane, the chemical constituents and bioactivities of *E. fischeriana* roots have been extensively studied and the known chemical constituents of this medicinal herb include monoterpenoids, flavonoids, anthraquinones, tannins, cerebrosides, glycerols, phenolics, diterpenoids, triterpenoids and steroids [15,16,17,18,19,20,21,22,23,24,25,26,27,28,29,30]. A number of abietane diterpenoids, including jolkinolide A and B, and 17-hydroxyjolkinolide A and B were isolated. Some of these diterpenoids exhibited significant antitumor activity against several tumor lines, such as prostate LNCaP, sarcoma 180, Ehrlich ascites carcinoma, and hepatocellar carcinoma in mice [31]. However, the bioactive compounds against insects have not been isolated and identified from this Chinese medicinal herb. In this paper, we report the isolation and identification of four feeding deterrents contained in *E. fischeriana* roots against two stored-product insects, *T. castaneum* and *S. zeamais* by bioassay-guided fractionation. 

## 2. Results and Discussion

### 2.1. Isolated bioactive compounds

**Figure 1:**
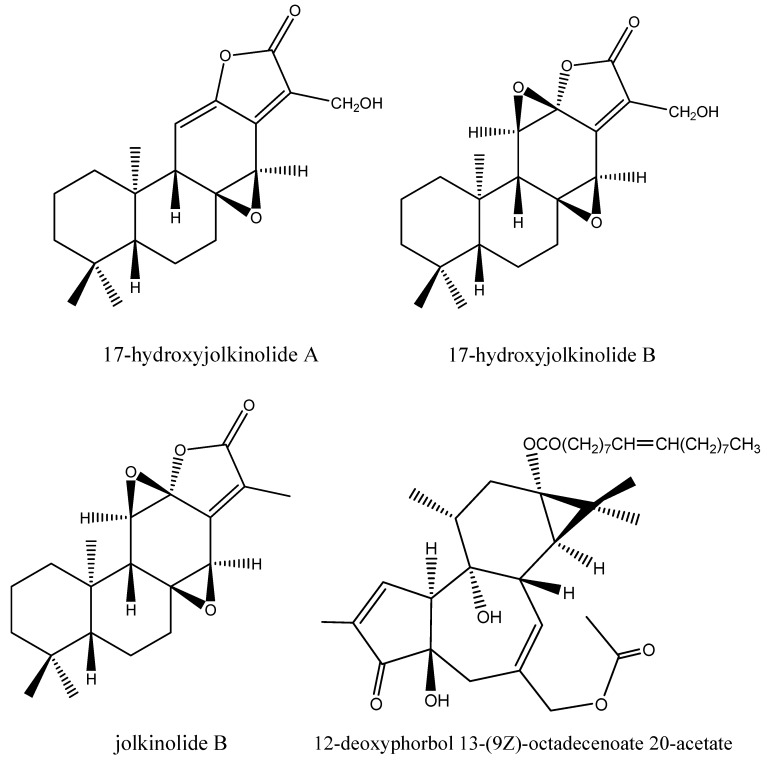
Structures of feeding deterrents isolated from *Euphorbia fischeriana* roots.

Four bioactive compounds were isolated and based on bioassay-guided fractionation and identified based on their spectroscopic data and comparison with literature vales. Their chemical structures are given in Figure 1.

### 2.2. Feeding deterrent activity

The feeding deterrent activity of the four isolated compounds against aize weevils and red flour beetles is shown in Table 1 and Table 2. Two of the pure compounds, namely 17-hydroxyjolkinolide B and jolkinolide B exhibited significant feeding deterrent activities against *T. castaneum* adults at a concentration of 30 ppm and above (Table 2). However, 17-hydroxyjolkinolide A and 12-deoxyphorbol 13-(9*Z*)-octadecenoate 20-acetate A possessed significant feeding deterrent activities against *T. castaneum* adults only at a concentration of 100 ppm and above (Table 2). Among the four isolated compounds, jolkinolide B showed strongest feeding deterent activities against *S. zeamais* and *T. castaneum* adults with EC_50_ values of 342.1 and 361.4 ppm, respectively. At the highest concentration (1,000 ppm), *S. zeamais* and *T. castaneum* adults consumed only 39.78% and 37.58% of diet contained jolkinolide B, respectively, compared with the control. However, 12-deoxyphorbol 13-(9*Z*)-octadecenoate 20-acetate possessed the least feeding deterrent activities against the two grain storage insects with EC_50_ values of 884.3 and 1058.4 ppm, respectively. For example, *S. zeamais* and *T. castaneum* adults consumed only 48.76% and 51.67% of diet contained jolkinolide B, respectively, compared with the control at the highest concentration (1,000 ppm).

Dietary 17 hydroxyjolkinolide A and B also exhibited feeding deterrent activity *S. zeamais* (EC_50 _= 631.9 and 543.9 ppm, respectively) and *T. castaneum* adults (EC_50 _= 656.6 and 551.5 ppm, respectively). When compared with the commercial feeding deterrents, azadirachtin and toosendanin, the four isolated compounds were less active against *S. zeamais* adults (EC_50_ values of azadirachtin and toosendanin were 57 and 100 ppm, respectively) and *T. castaneum* adults (EC_50_ values of azadirachtin and toosendanin were 3 and 66 ppm, respectively) [38,39].

**Table 1:** Feeding deterrent activities of the pure compounds isolated from *E. fischeriana* against *S. zeamais* adults.

Compounds	Concentration (ppm)	Consumption of diet* (% control ± SD)	EC_50_ (95% FL)	Slope±SD	Chi square (χ^2^)
Control		100.0 ± 4.87a	-	-	
17-Hydroxyjolkinolide A	1000	47.02 ± 2.05e			
	300	61.53 ± 2.91d	631.9	2.77 ± 0.15	48.42
	100	70.96 ± 1.85c	(567.2-697.4)		
	30	90.82 ± 3.34b			
	10	99.45 ± 2.17a			
17-Hydroxyjolkinolide B	1000	43.79 ± 3.89e			
	300	56.07 ± 3.73d	543.9	2.30 ± 0.10	17.49
	100	72.89 ± 2.62c	(494.7-603.8)		
	30	82.87 ± 2.83b			
	10	94.18 ± 3.45a			
Jolkinolide B	1000	39.78 ± 2.62e			
	300	48.57 ± 2.18d	342.1	2.23 ± 0.09	26.91
	100	63.84 ± 2.96c	(296.3-393.5)		
	30	81.01 ± 4.21b			
	10	94.23 ± 3.48a			
12-Deoxyphorbol 13-(9Z)-octadecenoate 20-acetate	1000	48.76 ± 1.89e			
	300	68.43 ± 2.85d	884.3	2.87 ± 0.12	15.08
	100	80.27 ± 2.56c	(787.3-965.4)		
	30	91.09 ± 2.66b			
	10	98.97 ± 1.65a			

* Multiple range test using Tukey’s test (P < 0.05). Within each compound, the same letters denote treatments not significantly different from each other.

**Table 2:** Feeding deterrent activities of the pure compounds isolated from *E. fischeriana* against *T. castaneum* adults.

Compounds	Concentration (ppm)	Consumption of diet* (% control ± SD)	EC_50_ (95% FL)	Slope±SD	Chi square (χ^2^)
Control		100.00 ± 5.32a	-	-	
17-Hydroxyjolkinolide A	1000	48.78 ± 4.45d			
	300	59.12 ± 4.09c	656.5	2.63 ± 0.15	30.42
	100	76.56 ± 5.23b	(593.4-703.5)		
	30	90.81 ± 4.34a			
	10	98.45 ± 1.96a			
17-Hydroxyjolkinolide B	1000	44.34 ± 5.56e			
	300	56.64 ± 3.73d	551.5	2.42 ± 0.13	17.60
	100	72.81 ± 4.21c	(489.3-606.5)		
	30	87.43 ± 5.38b			
	10	94.18 ± 3.45a			
Jolkinolide B	1000	37.58 ± 3.67e			
	300	49.78 ± 4.32d	361.4	2.24 ± 0.12	29.70
	100	66.43 ± 5.16c	(289.8-422.6)		
	30	80.31 ± 3.45b			
	10	96.23 ± 3.48a			
12-Deoxyphorbol 13-(9Z)-octadecenoate 20-acetate	1000	51.67 ± 4.93e			
	300	70.67 ± 3.23c	1058.4	1.96 ± 0.09	35.94
	100	83.33 ± 3.08d	(985.3-1145.7)		
	30	92.28 ± 3.34a			
	10	97.97 ± 2.65a			

* Multiple range test using Tukey’s test (P < 0.05). Within each compound, the same letters denote treatments not significantly different from each other.

The concentration used in this study to observe feeding deterrent effects was much higher than for the commercially available products such as margosan-O, active at a 3.75 ppm azadirachtin level [36]. However, it was comparable with another commercially product toosendanin at 20 ppm [37]. The four compounds were evaluated for feeding deterrent activity against stored product insects for the first time.

## 3. Experimental

### 3.1. Plant material

Dried roots (10 kg) of *E. fischeriana* were purchased from Anguo Chinese Medicinal Herbs Market (Anguo 071200, Hebei Province, China). The roots were ground to a powder using a grinding mill (Retsch Muhle, Germany). The species was identified, and the voucher specimens (BNU-CMH-Dushuahan-2009-08-25-004) were deposited at the Herbarium (BNU) of College of Life Sciences, Beijing Normal University.

### 3.2. Insects

The maize weevil, *S. zeamais* and red flour beetle, *T. castaneum* were obtained from laboratory cultures maintained for the last 10 years in the dark in incubators at 28-30 °C and 70-80% relative humidity. *T. castaneum* was reared on wheat flour mixed with yeast (10:1, w:w) while *S. zeamais* was reared on whole wheat at 12-13% moisture content. Adults of the two species used in all the experiments were about 2 weeks old.

### 3.3. Feeding deterrent activity

A flour disk bioassay was used to direct the isolation of active compounds from *E. fischeriana* roots according to the method of Xie *et al*. [36] with some modifications [4,38]. Wheat flour (1.0 g) was ultrasonically stirred in distilled water (5 mL) and ethanol (50 µL) containing a fraction or pure compound was added. Pure compounds were first dissolved in ethanol (500 μL) and two drops of Tween-20 (approximately 50 μg) were added to the wheat flour suspension. Aliquots (200 μL) of this stirred suspension were placed on the bottom of a polystyrene Petri dish to form disks. The pipette was fitted with a disposable tip that had an opening enlarged to about 2 mm internal diameter by cutting about 1 cm from the bottom of the tip with a razor blade. The same amounts of ethanol and Tween-20 were applied to produce the control flour disks. The flour disks were left in the fume-hood overnight to air dry. The flour disks were then transferred to an incubator to equilibrate at 28-30 °C and 70-80% R.H. for 48 h. Each flour disk weighed between 36 and 39 mg. The moisture content of the disk was determined to be 13.5 ± 0.1% using the Kett’s Grain moisture tester (Model PB-1D2, Japan). The disks were placed in glass vials (diameter 2.5 cm, height 5.5 cm) for weighing. Twenty group-weighed, unsexed insects were then added to each vial prior to further weighing. All the insects were starved for 24 h before use. Six replicates were carried out for all treatments and controls. The experimental set-up was left in the incubator for 3 days. Finally, the uneaten parts of the flour disks were weighed. The insect consumption for the different test substances was compared to the control group. Glass vials containing treated flour disks but without insects were prepared to determine any decrease in weights that might have occurred due to evaporation of solvents. 

### 3.4. Extraction and isolation of active ingredients

The powdered roots of *E. fischeriana* were extracted with 95% ethanol (50 L) at room temperature over a period of three weeks, and the extract was evaporated under reduced pressure using a vacuum rotary evaporator to afford a syrupy gum (256 g). This syrup was partitioned between methanol-water and petroleum ether (3 × 5,000 mL). The petroleum ether extracts were evaporated off to given a residue (38 g). The aqueous layer was re-partitioned with chloroform (3 × 5,000 mL) to provide a residue (173 g) after evaporation of chloroform. Further partitioning with ethyl acetate (3 × 5,000 mL) gave a residue (76 g) after evaporation of the solvent. The CHCl_3_ residue (25 g) was applied to a silica gel column (160-200 mesh, Qingdao Marine Chemical Plant, Shandong Province, China), eluting with petroleum ether containing increasing accounts of ethyl acetate (from 100:1 to 1:2) to give fourteen combined fractions according to TLC detection. Based on bioassay, Fraction 6, 8, 10 and 11 were chosen for further fractionation. Jolkonolide B (312 mg) was isolated from Fraction 6 (330 mg) after repeated purification on silica, Sephadex LH-20 (Pharmacia, Sweden) and PTLC (pre-coated GF254 plates, Qingdao Marine Chemical Plant). Fraction 8 (141 mg) was further chromatographed on silica gel column, Sephadex LH-20 as well as repeated PTLC to provide the bioactive compound which was recrystallized and determined to be 12-deoxyphorbol-13-(9*Z*)-octadecanoate-20-acetate (6.2 mg). 17-Hydroxyjolkinolide A (126.8 mg) was obtained from further chromatographed on silica gel TLC and Sephadex LH-20 and recrystallized from fraction 10 (409 mg). Fraction 11 (555 mg) was further chromatographed on silica gel column, Sephadex LH-20 and silica gel TLC to obtain the bioactive compound which was recrystallized and determined to be 17-hydroxyjolkinolide B (330 mg). The structures of the compounds were elucidated based on high-resolution electron impact mass spectrometry and nuclear magnetic resonance. 

### 3.5. Apparatus

Melting points were measured on a Buchi 535. ^1^H- and ^13^C-NMR spectra were recorded on a Bruker Avance DRX 500 instrument using CDCl_3_ as solvent with TMS as internal standard. EIMS were determined on an ThermoQuest Trace 2000 mass spectrometer at 70 eV (probe). 

### 3.6. Compound characterization

*17-Hydroxyjolkinolide* A. White needle-like crystals (CHCl_3_), m.p. 177-182 °C (m.p. 180-182 °C [24]), EI-MS m/z (%): 329 [M^+^] (5), 229 (10), 215 (11), 191 (12), 187 (22), 188 (20), 175 (58), 173 (100), 161 (19), 162 (24), 118 (17), 108 (20), 95 (23), 94 (38), 78 (45), 68 (53), 54 (42), 40 (55), C_20_H_26_O_4_. ^1^H-NMR (500 MHz) 5.60 (d, *J* = 4.6 Hz, 1H, H-11), 4.05 (s, 1H, H-14), 2.68 (d, *J* = 4.6 Hz, 1H, H-9), 4.65 (s, 2H, H-17), 0.97 (s, 3H, H-18), 0.88 (s, 3H, H-19), 0.76 (s, 3H, H-20). ^13^C-NMR (125 MHz) 169.2 (C-16), 147.3 (C-12), 146.6 (C-13), 127.3 (C-15), 106.5 (C-11), 61.3 (C-8), 56.5 (C-17), 54.4 (C-14), 53.5 (C-5), 51.8 (C-9), 41.5 (C-3), 41.3 (C-10), 39.9 (C-1), 34.0 (C-7), 33.6 (C-4), 33.4 (C-18), 21.9 (C-19), 20.8 (C-6), 18.4 (C-2), 14.1 (C-20). The ^1^H- and ^13^C-NMR data were in agreement with the reported data [28,31].

*17-Hydroxyjolkinolide* B. White needle-like crystals (CHCl_3_), m.p. 264-265 °C (m.p. 265 °C [31]), EI-MS m/z (%): 346 [M^+^] (3), 257 (12), 174 (13), 161 (14), 149 (18), 120 (24), 114 (32), 104 (38), 94 (43), 90 (66), 78 (63), 68 (65), 54 (82), 40 (100). C_20_H_26_O_5_. ^1^H-NMR (500 MHz) 4.70 (d, *J* = 12.0 Hz, 2H, H-17), 4.14 (s, 1H, H-14), 4.09 (s, 1H, H-11), 2.32 (s, 1H, H-9), 0.96 (s, 3H, H-18), 0.88 (s, 3H, H-19), 0.88 (s, 3H, H-20). ^13^C-NMR (125 MHz) 168.1 (C-16), 151.0 (C-13), 132.9 (C-15), 85.4 (C-12), 66.7 (C-8), 61.5 (C-11), 56.5 (C-17), 55.2 (C-14), 53.5 (C-5), 47.8 (C-9), 41.2 (C-3), 39.2 (C-10), 39.1 (C-1), 35.6 (C-7), 33.5 (C-4), 33.4 (C-18), 21.8 (C-19), 20.8 (C-6), 18.4 (C-2), 15.5 (C-20). The ^1^H- and ^13^C-NMR data were in agreement with the reported data [9,22,30].

*Jolkinolide* B. White needle-like crystals (CHCl_3_), m.p. 213-215 °C (m.p. 217 °C [28]), EI-MS m/z (%): 329 [M^+^] (4), 306 (14), 219 (15), 215 (25), 204 (26), 193 (30), 177 (36), 164 (38), 149 (40), 148 (98), 141 (72), 131 (37), 113 (35), 109 (94), 96 (100), 69 (43), 43 (37). C_20_H_26_O_4_. ^1^H-NMR (500 MHz) 4.07 (s, 1H, H-11), 3.71 (s, 1H, H-14), 2.32 (s, 1H, H-9), 2.11 (s, 3H, H-17), 0.97 (s, 3H, H-18), 0.88 (s, 3H, H-19), 0.85 (s, 3H, H-20). ^13^C-NMR (125 MHz) 169.0 (C-16), 148.6 (C-13), 130.2 (C-15), 85.2 (C-12), 66.0 (C-8), 60.9 (C-11), 55.3 (C-14), 53.4 (C-5), 48.0 (C-9), 41.2 (C-3), 39.2 (C-10), 39.1 (C-1), 35.6 (C-7), 33.5 (C-4), 33.4 (C-18), 21.8 (C-19), 20.8 (C-6), 18.4 (C-2), 15.4 (C-20), 8.75 (C-17). The ^1^H- and ^13^C-NMR data were in agreement with the reported data [32,35].

*12-Deoxyphorbol* *13-(9Z)-octadecenoate* *20-acetate.* White powder (CHCl_3_), EI-MS m/z (%): 654 [M^+^] (3), 337 (24), 311 (22), 313 (35), 239 (23), 237 (12), 149 (10), 134 (13), 123 (16), 108 (26), 97 (67), 69 (63), 55 (100). C_40_H_62_O_7_. ^1^H-NMR (500 MHz) 7.63 (1H, s, H-1), 5.73 (1H, d, *J* = 4.1Hz, H-7), 5.36 (2H, m, H-9¢ and H-10¢), 4.48 (2H, ABq, *J* = 12.3, 7.3 Hz, H-20), 3.30 (1H, brd, *J =* 2.2 Hz, H-10), 3.02 (1H, t, *J* = 5.1 Hz, H-8), 2.02 (3H, s, OAc-20). ^13^C-NMR (125 MHz) 209.1 (C-3), 161.4 (C-1), 135.0 (C-6), 133.8 (C-7), 132.8 (C-2), 75.9 (C-9), 73.6 (C-4), 69.4 (C-20), 63.2 (C-13), 55.7 (C-10), 39.4 (C-8), 38.9 (C-5), 36.3 (C-11), 32.4 (C-14), 31.9 (C-12), 23.2 (C-16), 22.6 (C-15), 18.5 (C-18), 15.3 (C-17), 10.0 (C-19), 175.9 (C-1¢), 34.2 (C-2¢), 24.9 (C-3¢), 28.9-29.8 (C-4¢-C-8¢ and C-12¢-C-15¢), 31.8 (C-16¢), 22.6 (C-17¢), 14.1 (C-18¢), 173.4 and 21.2 (OAc-20). The ^1^H- and ^13^C-NMR data were in agreement with the reported data [29]. 

### 3.7. Data analyses

Analysis of variance (ANOVA) and Tukey’s test were conducted by using SPSS 10 for Windows 98. Percentage was subjected to an arcsine square-root transformation before ANOVA and Tukey’s tests. The EC_50_ (the concentration needed to inhibit insect feeding by 50% relative to controls) was determined by linear regression (40).

## 4. Conclusions

Based on mass screening of medicinal herbs, the ethanol extract of *E. fischeriana* roots was found to possess feeding deterrent activity against the two grain storage insects, the red flour beetles (*T. castaneum*) and maize weevil (*S. zeamais*). Four feeding deterrents were isolated and identified from the ethanol extract of *E. fischeriana* roots by bioactivity-guided fractionation. The concentration used in this study to observe feeding deterrent effects was comparable with the commercially product toosendanin. These findings suggest that the ethanol extract of *E. fischeriana* roots and four isolated compound show potential for development as natural feeding deterrent fumigants for stored products.
